# Time Is Money: Considerations for Measuring the Radiological Reading Time

**DOI:** 10.3390/jimaging8080208

**Published:** 2022-07-24

**Authors:** Raphael Sexauer, Caroline Bestler

**Affiliations:** Department of Radiology and Nuclear Medicine, University Hospital Basel, 4031 Basel, Switzerland; caroline.bestler@usb.ch

**Keywords:** economic, reporting, time stamps, radiology, modelling, outlier detection

## Abstract

Timestamps in the Radiology Information System (RIS) are a readily available and valuable source of information with increasing significance, among others, due to the current focus on the clinical impact of artificial intelligence applications. We aimed to evaluate timestamp-based radiological dictation time, introduce timestamp modeling techniques, and compare those with prospective measured reporting. Dictation time was calculated from RIS timestamps between 05/2010 and 01/2021 at our institution (*n* = 108,310). We minimized contextual outliers by simulating the raw data by iteration (1000, vector size (µ/sd/λ) = 100/loop), assuming normally distributed reporting times. In addition, 329 reporting times were prospectively measured by two radiologists (1 and 4 years of experience). Altogether, 106,127 of 108,310 exams were included after simulation, with a mean dictation time of 16.62 min. Mean dictation time was 16.05 min head CT (44,743/45,596), 15.84 min for chest CT (32,797/33,381), 17.92 min for abdominal CT (*n* = 22,805/23,483), 10.96 min for CT foot (*n* = 937/958), 9.14 min for lumbar spine (881/892), 8.83 min for shoulder (409/436), 8.83 min for CT wrist (1201/1322), and 39.20 min for a polytrauma patient (2127/2242), without a significant difference to the prospective reporting times. In conclusion, timestamp analysis is useful to measure current reporting practice, whereas body-region and radiological experience are confounders. This could aid in cost–benefit assessments of workflow changes (e.g., AI implementation).

## 1. Introduction

A scientifically rigorous and valid performance measurement is essential for quality improvement and therefore health care [1,2]. The art of reporting is currently in a state of upheaval, and various recent developments are predicted to revolutionize radiology.

While an increasing implementation of machine learning algorithms is expected for radiological reporting due to improving performance, various roadblocks still hamper broad clinical implementation [3]. Besides diagnostic accuracy, acceptance by human experts is crucial to improve accountability of Computer-aided Diagnosis (CAD) Systems [4]. Herein, the human–computer interaction in clinical decision support systems plays an important role and must be included to assess efficiency [5]. A recently published survey by the European Society of Radiology (ESR) showed that many AI algorithms do not meet clinical expectations, and that higher workloads compared to their added value prevent their implementation [6]. Considering increasing workloads, the current discussion of reporting format with varying degrees of structuring, multimedia enhancement, and the implementation of IT solutions might also increase radiological reading time. 

With suitable measuring points of the current structures, processes, and the associated treatment outcome, weak points can be identified and compared with possible solution strategies [7]. The goal is to increase quality and reduce existing costs in parallel [8]. Therefore, it is necessary to record the actual radiological reading time and analyze the influence of new approaches on them. With the current common use of speech recognition in radiological reporting and the use of Radiology Information System (RIS) systems, timestamps are routinely and automatically recorded and are a valuable source of information [9]. However, these are susceptible to contextual outliers like interruptions within the report, which can both extend and shorten the registered time. An urgent phone call can lead to a shortened time entry through immediate caching, literature research, or conversations while the report is still open, and can additionally lead to a delayed time entry.

The aim of the study is to assess the radiological reading time based on Speech-Recognition related RIS timestamps, reduce systematic outlier via simulation based on the expected normal distribution, and validate the estimates with prospectively recorded reporting time.

## 2. Materials and Methods

### 2.1. Data

The Radiology Information System (Centricity RIS-i 7, 7.0.1.7; General Electric Company, Boston, MA, USA) automatically saves the time of dictation start as well as the time of the first saving. In our current practice, we routinely use (anatomically) structured reporting, combined with the use of speech recognition (Philips SpeechMike III Pro Premium LFH3500, Philips, Amsterdam, The Netherlands). We screened for all recorded timestamps for head CTs (*n* = 45,596), chest CTs (*n* = 33,381), abdominal CTs (*n* = 23,483), foot CTs (*n* = 958), lumbar spine CTs (*n* = 892), shoulder CTs (*n* = 436), wrist CTs (*n* = 1322), and polytrauma CTs (*n* = 2242) between 05/2010 and 01/2021 where a speech recognition was used. To avoid inappropriate exclusion and for simplification, different protocols, including multiphase imaging, were pooled according to the specific anatomical subsite. For example, head CT ranges for CTs without contrast to Stroke-protocols (native, CTA, Perfusion imaging).

The examination entity and the times for the start of the speech recognition and the first saving of the report were exported. 

### 2.2. Definitions

For comprehension, we specified RIS–timestamps-based radiological reading time as dictation time and the prospective measured reading as reporting time throughout the manuscript. We defined the time between dictation start and first saving as dictation time. Cases in which the reports were first saved before the dictation was started were excluded. 

### 2.3. Simulation for Outlier Detection

We defined outliers as data points that deviate significantly from the expected distribution. Since comorbidities influence the radiological reading time, there is a mean, individual reporting time in daily practice which shows a certain scatter range due to varying case complexity, whereas extremes are characterized as less likely (e.g., 2 min or 90 min for CT abdomen). Based on indicative values from the literature and clinical experience, four independent variables were identified: too short time entries due to interruptions for caching or release (1 = interrupts), true radiological reading (2: reporting time), too long time entries due to interruptions without buffering but the continuation of reporting to a later time point (e.g., overnights), as well as time entries of unknown cause (4). 

Since in opposite to the other indicative values, true radiological reading best fits a normal distribution, we developed a mathematical simulation (R 3.4.3) based on the expectation-maximization algorithm to minimize outliers.

By simulating normally distributed values, lambda, mean, and standard deviation were iteratively optimized for the simulation. We chose 1000 iterations with a respective vector size of 100 values for lambda, mean, and standard deviation, which were adapted with increasing iteration steps. A simulation was accepted when the mean, standard deviation, and lambda approached a specific value as the iteration increased. The code of the simulation can be found in Appendix A.

### 2.4. Real Reporting Time

As for validation, we aim to compare the reporting time means with the dictation time using a two-tailed *t*-test. A random sample of 10 measured reporting times (reader 1: 4 years of experience) with a mean of 13.3 min and a standard deviation of 4.2 min was used for the sample size estimation, based on the following Equation [10]:

(1) $$N=\frac{4\sigma^{2}{\left(z_{crit}+z_{pwr}\right)}^{2}}{D^{2}}$$

N = total sample size, *σ* = assumed SD, *z_crit_* and *z_pwr_* = Standard normal deviates (for the significance criterion and the Statistical Power), *D* = total width of the expected CI. 

For a power of 0.9 (*z_pwr_* = 1.282) and 0.05 as significance criterion (*z_crit_* = 1.96), a minimum of 329 reporting times (N) is needed to detect a minimum difference of 1.5 min (D). Therefore, two radiologists (reader 1, reader 2: 1 year of experience) prospectively measured their real-time reporting between 01/2021 and 12/2021 for head CT, chest CT, abdominal CT, foot CT, lumbar spine CT, shoulder CT, wrist CT, and polytrauma CT. Interruptions were excluded.

### 2.5. Statistics

The descriptive statistics were represented by mean, standard deviation, median, skewness, and kurtosis. We used Kolmogorov–Smirnov to test for normal distribution. For differences in mean reporting time *t*-test was performed, and Mann-Whitney-U-Test to compare case complexity tendencies between first and second prospective assessment period. The simulations were performed for head CT, chest CT, abdominal CT, foot CT, lumbar spine CT, shoulder CT, wrist CT, and polytrauma CT. A *p*-value of <0.05 was considered statistically significant. All statistical analyses were performed with R 4.0.5 (R Core Team, Vienna, Austria).

## 3. Results

### 3.1. Reporting Time

**Figure 1:**
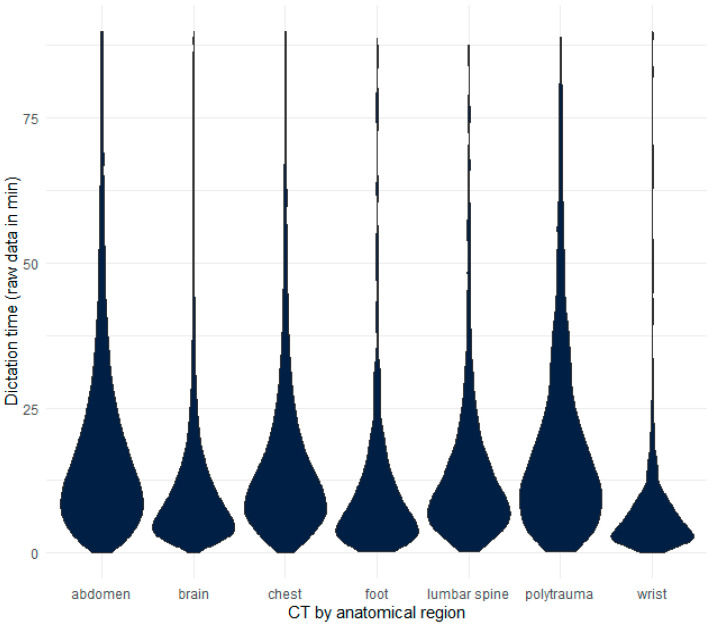
Violin chart shows all CT dictation times by anatomical regions from 05/2010 until 01/2021 before simulation for outlier reduction. For better clarity, longer time entries in the graph (>80 min) were not taken into account. Note that the charts seem to be truncated, which is caused by outliers with short time entries, e.g., by initially caching the report after starting the dictation software. Nevertheless, the data seem to be normally distributed, which can only be explained by the proportion of the actual radiological reading time.

Before simulation for outlier detection, CT abdomen, foot, lumbar spine, wrist, and polytrauma were normally distributed (Kolmogorov–Smirnov: *p* > 0.05). The initial median dictation time for head, chest, abdomen, foot, lumbar spine, wrist, and polytrauma CT was head 8.94 min (mean: 27.24 min, SD: 65.53 min), chest 13.42 min (mean: 36.96 min, SD: 72.83 min), 16.00 min (mean: 40.74 min, SD: 76.32 min), 8.08 min (mean: 24.54 min, SD: 52.68 min), 11.63 min (mean: 37.85 min, SD: 79.55 min), 5.68 min (mean: 19.98 min, SD: 51.13 min), and 15.80 min (mean: 37.07 min, SD: 74.08 min), respectively, as shown in Figure 1. 

The results of simulation are summarized in Table 1. Note the consistency between mean and median. There is a large variance in case complexity and radiological experience (multiple observers) impacting the standard deviation.

#### Real Reporting Time

**Figure 2:**
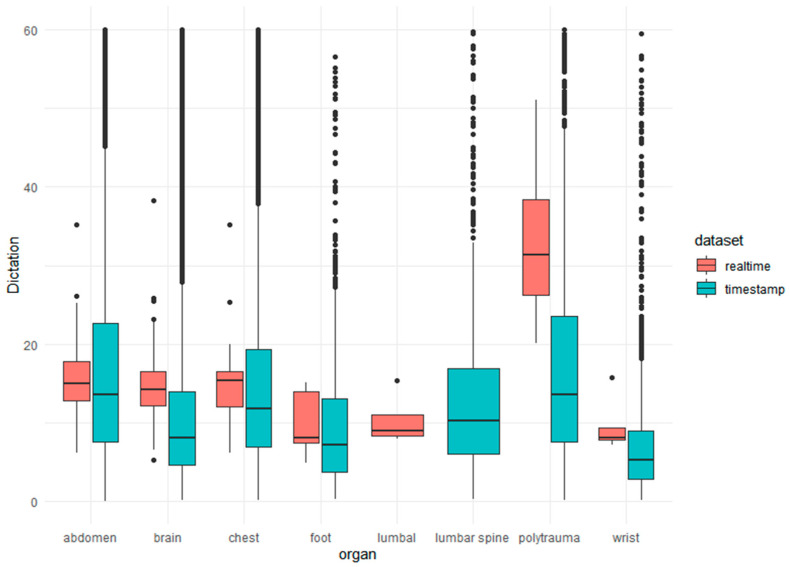
The box plots compare the prospective 329 real-reporting times (red) with the timestamps of the 108,310 examinations (light blue) before simulation for outlier reduction.

In total, the time of 329 reporting times was recorded prospectively. The mean reporting time was 16.01 min (SD: 7.48 min, 95% CI: 15.20–16.83 min, Median 15.00 min). The reporting time for head CT (*n* = 135), chest CT (*n* = 95), abdominal CT (*n* = 57), foot CT (*n* = 7), lumbar spine CT (*n* = 6), wrist CT (*n* = 5), and polytrauma CT (*n* = 26) were 14.90 min (SD: 5.66 min), 14.38 min (SD: 5.34 min), 15.84 min (SD: 5.17 min), 10.13 min (SD: 5.45 min), 10.08 min (SD: 2.68 min), 9.66 min (SD: 3.53 min), and 32.16 min (SD: 8.74 min). However, reporting times for abdominal, foot, lumbar spine, wrist, and polytrauma CT were normally distributed (Kolmogorov–Smirnov, *p* > 0.05), and measures for head and chest CT showed a relative high kurtosis with 3.41 (standard error: 0.41) and 2.81 (standard error: 0.50). The corresponding skewness was 1.40 (standard error: 0.21) and 0.97 (standard error: 0.25). Figure 2 compares real-time reporting time with the acquired dictation time based on timestamps. There is no significant difference between the simulation and the real-time subset (*p* < 0.001). Experience had an influence on the mean reporting time (*p* = 0.004): reader 1 required a mean of 15.47 min (SD: 7.15 min) and reader 2 required a mean of 19.83 min (SD: 8.71 min). Within the observation period, there was no significant reduction (*p* = 0.351) in individual reporting time. For example, reader 1 required 15.08 min in the first period versus 15.87 min in the second, with a slight increase, but not significantly more time-consuming reports in the second period (*p*: 0.07).

## 4. Discussion

An iterative simulation of dictation time based on RIS timestamps can be used to reduce systemic outliers and allows monitoring of radiological reading time on a large database if speech recognition is used. The simulated dictation time was, on average, 16.62 min without a significant time difference to a small sample of 329 real-time reports.

The turnaround time is established to monitor changes in the work process, e.g., changing the reporting system from decentralized/modality-based to centralized/subspecialized can result in a reduced report turnaround time (RTAT) [11]. However, the turnaround time corresponds to the actual effective working time to a limited extent. If the workload is high for two radiologists who work fast, and both are involved in the preparation of the report, the turnaround time may be high, despite the high level of effectiveness. Conversely, if the workload is low, the turnaround time is shortened, despite the low effectiveness. 

To address this problem, McDonald proposed a timestamp approach to more accurately measure reporting time [12]. However, these are outlier-prone. For example, to allow fast-track in stroke patients (door to needle) neuroradiologists are on-call, and interrupt routine reporting to facilitate immediate decision-making at the CT-workstation, which might lead to a “false-long” time registries. Therefore, McDonald et al. used 60 min as an upper cutoff per CT report and only reported medians in their study (more robust for a skewed distribution) and lacked a prospective real-time validation. Their measured medians largely correspond to our mean values after outlier reduction. They measured a median duration (minutes) of 15.21 min for chest (*n* = 2469), 14.34 min for abdomen (*n* = 5710), 13.15 min for lumbar spine (*n* = 102), and 32.36 min for polytrauma (*n* = 122), which is comparable to our data (chest: 15.84 min, *n* = 32,797; abdomen: 17.92 min, *n* = 22,805; foot: 10.96 min, *n* = 937; lumbar spine: 9.14 min, *n* = 881; polytrauma: 39.2 min, *n* = 2127). 

However, reporting time also has a natural variance. Reporting time will also be extended in difficult cases in need of case discussion with colleagues, literature research, or lack of clinical information. 

Therefore, simulation for outlier reduction seems to be reasonable. This allows for the assessment of workload changes using statistical tests based on mean values or normal distribution. Using this method, even smaller samples can be compared with the institution’s own generated reference reporting time after introducing a change in the workflow. This might be useful in assessing changes in the radiology report format [13], the implementation of artificial intelligence [14], and analyses of confounding factors such as fatigue [15]. 

There are some limitations to this study. The recorded timestamps were not recorded for the purpose of the study and the prospectively collected evaluation data set is relatively small. However, since most RIS with integrated speech recognition use the timestamps described herein, in our opinion this increases the reproducibility of our study. Another limitation is the assumption of a normal distribution for the reporting time. However, in comparison to the use of simple cutoffs, this can reduce an a priori exclusion of plausible long reporting times in difficult cases which, e.g., require discussion with colleagues or literature research. Additionally, the radiological reading times might be unique to our department due to complex tertiary level cases (e.g., postoperative head CT). Nevertheless, this study’s aim is not to compare absolute times to other institutions, but rather to provide a method to quantify and compare reporting times. The evaluated times in this study do not represent the total workload of a radiologist. Rather, they provide approaches to estimate the reporting time based on dictation time entries of routine cases, especially since the time of the first viewing of the images might differ from the start of the dictation. Lastly, recorded timestamps are dependent on the software used and data availability is dependent on the Graphical User Interface (GUI). However, GE provides features common in alternative providers. 

In summary, timestamp analyses enable large sample-size analysis for cost–benefit analysis, but harper contextual outliers, where a simulation by normal distribution is feasible to improve data quality. Experience and anatomical region were identified as cofounders of reporting time. 

In future research, we would like to use the timestamp analyses prospectively for workflow changes. Specifically, we want to introduce different deep learning applications for cardiothoracic imaging [16,17] as well as multimedia enhanced reports (with hyperlinks to the image findings) and investigate their influence on the current reporting time.

## 5. Conclusions

Simulating the reporting time by a normal distribution can minimize contextual outliers like interruptions. This enables a robust estimate of routine reporting time with no extra costs based on large sample sizes.

## Figures and Tables

**Table 1:** *n* (total): all available timestamps, *n* (norm): cases after outlier reduction which follows a normal distribution.

	*n* (Total)	*n* (Norm)	Mean	Standard Deviation	Median
Head	45,596	44,743	16.05	31.27	16.37
Chest	33,381	32,797	15.84	30.21	16.16
Abdomen	23,483	22,805	17.92	31.95	17.75
Foot	958	937	10.96	20.16	10.80
Lumbar spine	892	881	9.14	13.27	8.91
Wrist	1322	1201	8.83	12.83	8.44
Polytrauma	2242	2127	39.2	52.41	39.36
All	107,874	105,491	16.62	33.11	16.58

## Data Availability

The data presented in this study are openly available in Zenodo at https://doi.org/10.5281/zenodo.6536195 (accessed on 22 July 2022).

## Appendix A

R Script (the following code can be used for modelling of timestamps assuming a normal distribution):

		 #data= your timestamps
		 N<-length(data)
		 t_max<-max(data)
		 ## function,to optimize:
		 optL <- function(l.vec, m, s)
		  {
		   error <- rep(NA, length(l.vec))
		   for(i in c(1:length(l.vec)))
		     {
		     sim.df <- data.frame(x1 = rnorm(N,mean=m, sd=s), x2 = rexp(N, l.vec[i]))
		     sim <- apply(sim.df, 1, min)
		     # plot(density(sim))
		     error[i] <- sum((sort(data) - sort(sim))^2)
		     }
		   return(l.vec[which.min(error)])
		   print(l.vec)
		  }
		  
		 ## function to optimize m 
		 optM <- function(m.vec,l,s)
		  {
		   error <- rep(NA,length(m.vec))
		   for(i in c(1:length(m.vec)))
		     {
		     sim.df <- data.frame(x1=rnorm(N,m.vec[i]),sd=s, x2 = rexp(N, l))
		     sim <- apply(sim.df,1,min)
		     error[i] <- sum((sort(data)- sort(sim))^2)
		     }
		   return(m.vec[which.min(error)])
		   print(m.vec)
		  }
		 ## function to optimize SD
		 optS <- function(m,l,s.vec)
		  {
		   error <- rep(NA,length(s.vec))
		   for(i in c(1:length(s.vec)))
		     {
		     sim.df <- data.frame(x1=rnorm(N,m,sd=s.vec[i]), x2= rexp(N,l))
		     sim <- apply(sim.df,1,min)
		     error [i] <- sum((sort(data)-sort(sim))^2)
		     }
		   return(s.vec[which.min(error)])
		   print(s.vec)
		  }
		  
		 ## Define range for search vector for l,m,s: 
		 my.vec <- function(x,i){
		  x.min <- x-(x/(i^2))
		  x.max <- x+(x/(i^2))
		  my.vec <-seq(from = x.min, to = x.max, length.out = 100)
		  return(my.vec)
		 }
		 ### prepare iteration
		 ## set variables: m,s,l, i=iteration step
		 m<-30
		 s<-10
		 l<-0.03
		 #set.seed= reproducibility
		 set.seed(50)
		 ###iteration start:
		 for(i in c(1:1500)){
		  ## optimize l with m, s 
		 my.l <- my.vec(l, i)
		 my.l <- my.l[my.l >0 and my.l < 1]
		 l <- optL(my.l,m, s)
		 ## optimize m with l, s
		 my.m <- my.vec(m, i)
		 my.m <- my.m[my.m >0 and my.m < 120]
		 m <- optM(my.m,l = l, s = s)
		 ## optimize s with l,m
		 my.s <- my.vec(s, i)
		 my.s <- my.s[my.s >0 and my.s < 120]
		 s <- optS(my.s,l = l, m = m)
		 ## plot each 100 iteration
		 if(i %% 100 == 0){
		  sim.df <- data.frame(x1=rnorm(N,m,sd=s), x2= rexp(N,l))
		  sim <- apply(sim.df,1,min)
		  norm <- sum(sim.df[, 1] == sim)
		  error <- sum((sort(sim) - sort(data))^2)
		  mypath<-file.path("D:/expo/PETCT/",paste("histogram_iteration_",i,".jpg",sep=""))
		  jpeg(file=mypath)
		  hist(sim, 
		     main = paste("Iteration", i,"von N(my,s):", norm,", m =", sprintf("%.2f", m), ", s =", sprintf("%.2f", s), ", l =", sprintf("%.2f",l), "error = ", error), 
		     xlim = c(0,t_max/3), ylim = c(0,t_max/3), 
		     col = "blue",
		     breaks=300,xlab="time(min)",ylab="frequency")
		  par(new = TRUE)
		  hist(data,
		     main = "",
		     xlim = c(0,t_max/3), ylim = c(0, t_max/3),
		     breaks = 300,xlab="",ylab="")
		  dev.off()
		  }
		 }
		 write.table(
		  paste("medium:",m,
		    "n(total):",N,
		    "n(norm):",norm,
		    "standard deviation:",s,
		    "lambda exponential:",l,
		    "iteration:",i),"D:/expo/PETCT/results/information.txt",sep="\t")
		 library(truncnorm)
		 x <- rtruncnorm(n = 118, a = 0, b = Inf, mean = m, sd = s)
		 y<-summary(sim.df)
		
